# Giant Gastric Trichobezoar: A Case Report of an Adolescent Girl

**DOI:** 10.1002/ccr3.71116

**Published:** 2025-10-03

**Authors:** Sehrish Irshad, Shahzeen Irshad, Maryam Irshad, Mahnoor Fatima, Zulqarnain Ali

**Affiliations:** ^1^ Nishtar Medical University Multan Pakistan; ^2^ Shaheed Ziaur Rahman Medical College Bogura Bangladesh

**Keywords:** case report, intragastric mass, trichobezoar, trichophagia, trichotillomania

## Abstract

A rare case of gastric trichobezoar associated with trichotillomania and trichophagia emphasizes the need for careful psychiatric evaluation and early imaging of such patients to ensure timely surgical intervention to prevent severe complications.

## 1 Introduction

Bezoars are compact masses formed by the accumulation of matter and are broadly divided into trichobezoars, phytobezoars, pharmacobezoars, and lactobezoars [1]. Trichobezoars are rare gastrointestinal masses composed of ingested hair, predominantly affecting pediatric and adolescent populations, though adult cases have been documented. They are often associated with psychiatric conditions such as trichotillomania, trichophagia, and obsessive compulsive disorder. The prevalence of trichobezoars is estimated to range from 0.06% to 4% in the general population, with a higher incidence observed in young females [2].

Trichobezoars occur typically among the mucosal folds of the stomach due to inadequate digestive breakdown of hair and delayed gastric emptying. In Rapunzel syndrome, a tail‐like extension of a hairy mass extends from the stomach to the intestine [3]. Clinically, trichobezoars present with nonspecific symptoms including abdominal pain, nausea, vomiting, and, in severe cases, may cause intestinal obstruction, ulceration, perforation, bleeding, protein‐losing enteropathy, and pancreatitis. Diagnosis can be challenging due to these vague symptoms and the rarity of the condition. Imaging modalities such as ultrasound, computed tomography (CT) scans, and endoscopy are instrumental in identifying these masses.

Surgical removal remains the primary treatment modality, as endoscopic methods are often ineffective due to the dense and large nature of trichobezoars. Postoperative management should include psychiatric evaluation and intervention to address underlying behavioral disorders and prevent recurrence [3].

This case discusses the diagnostic challenges and successful surgical management of a rare gastric trichobezoar in an adolescent patient, highlighting the importance of a multidisciplinary approach in such cases.

## 2 Case Image

A 13‐year‐old girl presented with a 6‐month history of intermittent abdominal pain and 1‐month history of repetitive nausea and vomiting of ingested food, early satiety, and weight loss. On physical examination, the patient was thin, with few bald spots on her head, and pallor was present. Abdominal examination revealed epigastric tenderness, a palpable, firm mass in the left upper abdomen, mild splenomegaly, and ascites. Laboratory investigations were unremarkable except for iron deficiency anemia, with a hemoglobin level of 7.4 g/dL.

**FIGURE 1:**
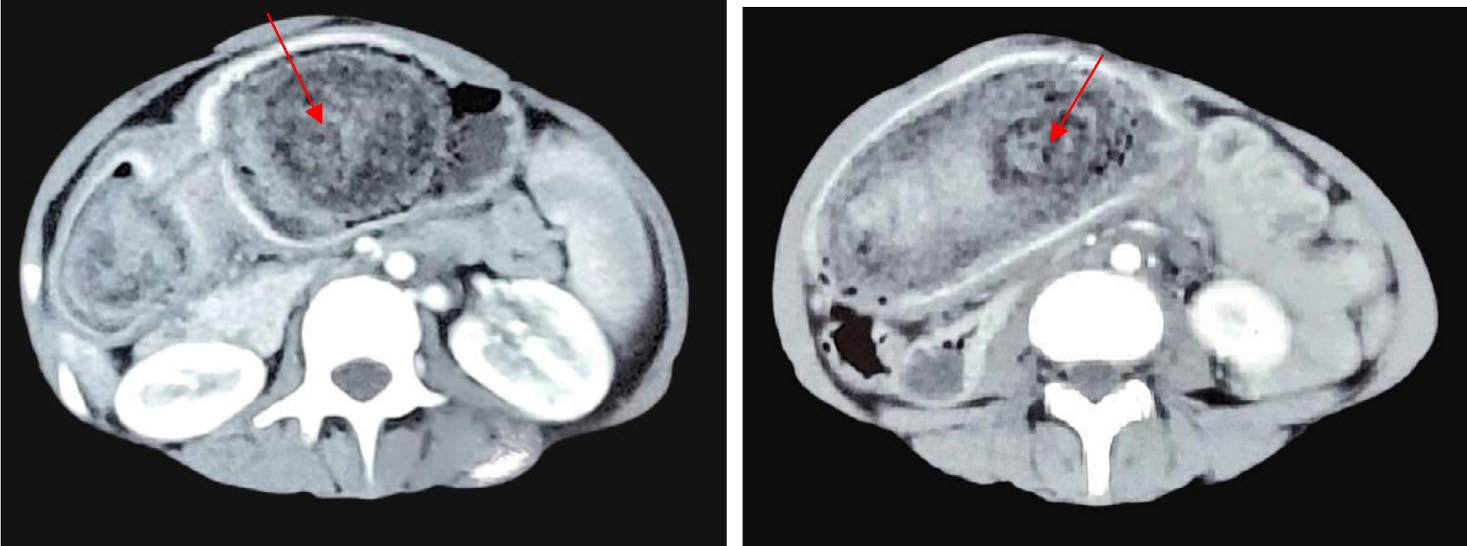
Contrast enhanced CT imaging of a gastric trichobezoar: Axial views demonstrating intraluminal mass with mottled gas pattern (shown by red arrows).

An upper gastrointestinal endoscopy under anesthesia revealed a large hair mass starting just below the lower end of the esophagus up to the pylorus. A CT scan of the abdomen revealed a well‐circumscribed, non‐enhancing, intra‐gastric mass with a mottled gas pattern or compressed concentric ring pattern due to the presence of entrapped hair and food debris extending into the pylorus, representing gastric bezoars along with mesenteric lymphadenopathy (Figure 1).

Upon further inquiry, the mother revealed that she had noticed her child eating and plucking her own hair. Psychiatric evaluation diagnosed her with trichotillomania and trichophagia. She was referred to a psychiatric clinic, and surgical intervention was scheduled after 3 weeks of psychiatric stabilization.

**FIGURE 2:**
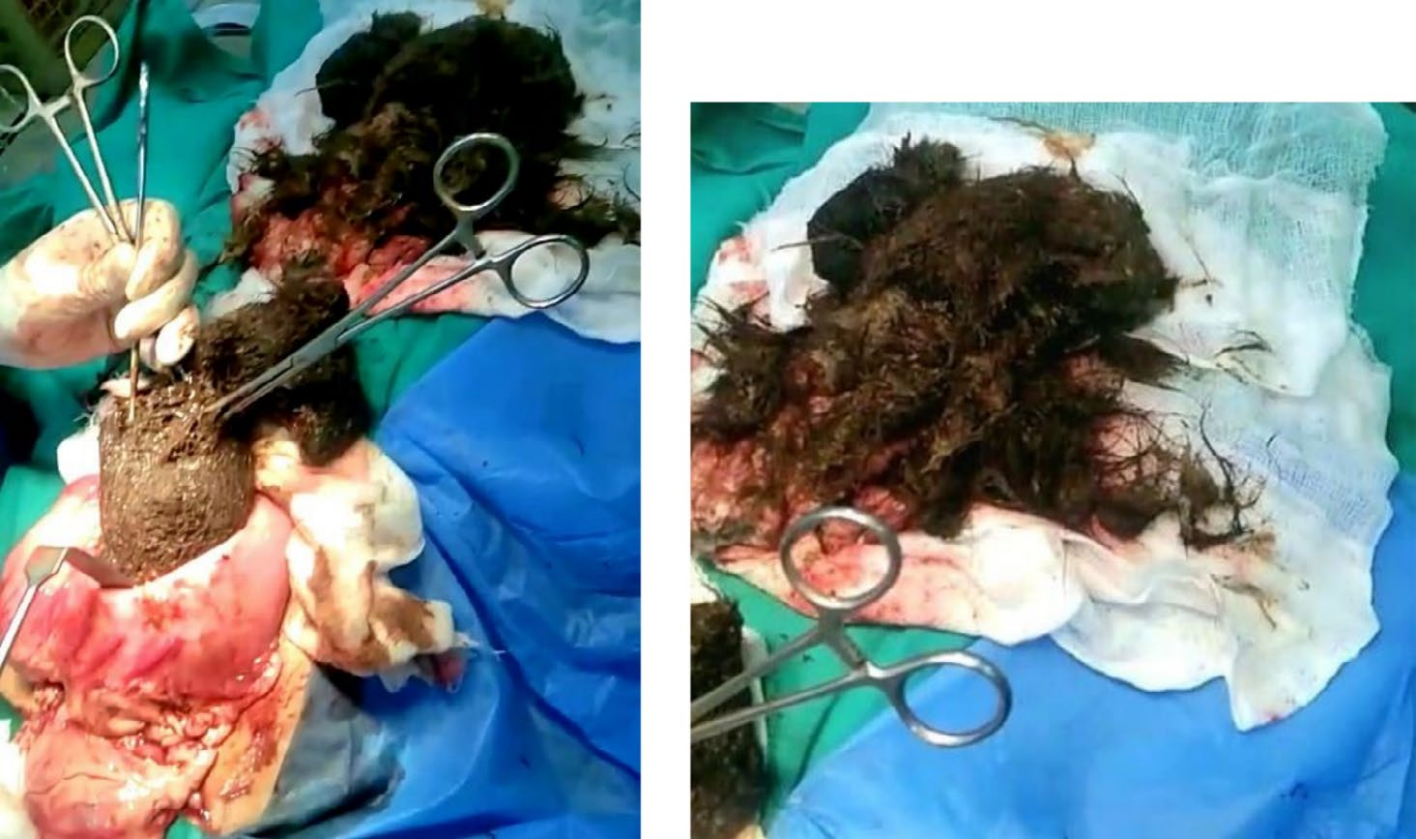
Intraoperative images demonstrating the removal of a large trichobezoar from the stomach through an open gastrotomy incision.

The patient underwent exploratory laparotomy and subsequent gastrostomy, extracting a huge trichobezoar in its entirety, measuring 25 × 15 cm extending from the lower end of the esophagus to the pylorus (Figure 2). A nasoduodenal tube was placed for postoperative feeding. The postoperative course was uneventful, and the patient was discharged with recommendations for ongoing psychiatric follow‐up.

## Author Contributions

**Sehrish Irshad:** conceptualization, methodology, project administration, supervision, writing – original draft, writing – review and editing. **Shahzeen Irshad:** conceptualization, data curation, investigation, writing – original draft. **Maryam Irshad:** conceptualization, data curation, investigation, writing – original draft. **Mahnoor Fatima:** conceptualization, data curation, investigation, writing – original draft. **Zulqarnain Ali:** conceptualization, writing – review and editing.

## Consent

Written informed consent for the publication of the patient's clinical information and imaging was obtained from the patient as well as the patient's guardian.

## Conflicts of Interest

The authors declare no conflicts of interest.

## Data Availability

The authors have nothing to report.
